# Skyglow-Induced Luminance Gradients Influence Orientation in a Migratory Moth

**DOI:** 10.3390/insects16121252

**Published:** 2025-12-10

**Authors:** Yi Ji, Yibo Ma, Zhangsu Wen, Boya Gao, James J. Foster, Daihong Yu, Yan Wu, Guijun Wan, Gao Hu

**Affiliations:** 1 State Key Laboratory of Agricultural and Forestry Biosecurity, College of Plant Protection, Nanjing Agricultural University, Nanjing 210095, China; jiyi@stu.njau.edu.cn (Y.J.); 2022102060@stu.njau.edu.cn (Y.M.); 2024102075@stu.njau.edu.cn (Z.W.); t2021101@njau.edu.cn (B.G.); hugao@njau.edu.cn (G.H.); 2 Centre for the Advanced Study of Collective Behaviour, University of Konstanz, 78464 Konstanz, Germany; james.foster@uni-konstanz.de; 3 Department of Biology, University of Konstanz, 78464 Konstanz, Germany; 4 Plant Protection Station of Yuanjiang County, Yuxi 653100, China; yudaihongyu@163.com; 5 Guizhou Key Laboratory of Agricultural Biosecurity, Guiyang University, Guiyang 550005, China; 15150535703@163.com

**Keywords:** fall armyworm, skyglow, orientation, light pollution, migratory insects

## Abstract

Artificial light at night (ALAN) is altering nocturnal ecosystems. While the effects of direct light sources on insect behavior are well studied, the influence of large-scale skyglow on migratory orientation remains unclear. Here, we tested how skyglow-induced luminance gradients influence the flight orientation of the fall armyworm, *Spodoptera frugiperda*, a globally invasive nocturnal migrant that performs seasonal migration in China, using controlled indoor simulations and field assays. Surprisingly, individuals consistently oriented toward darker regions, suggesting that luminance gradients may influence their heading away from the expected seasonal migratory direction. This response was highly consistent across both settings, indicating that skyglow-generated luminance gradients can function as directional cues and potentially interfere with seasonal orientation processes. Such gradients may thus function as ecological traps and represent an underrecognized factor in nocturnal insect navigation. Our findings point to a previously overlooked pathway through which skyglow may affect long-distance orientation in nocturnal migrants, underscoring the need for further work to evaluate its ecological significance within light-polluted environments.

## Simple Summary

Artificial light at night is spreading worldwide and changing the behavior of nocturnal animals. We studied how the fall armyworm, a migratory moth, responds to light pollution using indoor experiments and field observations. We found that moths consistently flew toward darker parts of the sky, even when this conflicted with their natural migratory direction. This suggests that skyglow can mislead insects, drawing them away from their normal orientation and trapping them in unsuitable habitats. At the same time, our results indicate that natural patterns of brightness in the night sky may help insects find their way. Reducing skyglow could therefore be important for protecting nocturnal biodiversity.

## 1. Introduction

Over the past century, the widespread adoption of electric lighting has transformed artificial light at night (ALAN) into a rapidly expanding environmental stressor worldwide [1,2]. By altering the intensity, duration, spectral composition, and polarization of natural darkness [3], ALAN disrupts nocturnal light cycles [4,5] and obscures celestial visual cues essential for orientation [6]. Unlike its seemingly two-dimensional appearance in satellite imagery, ALAN is a three-dimensional phenomenon: light is emitted, reflected, and scattered across altitudes and angles, reaching even habitats not directly illuminated [7]. A particularly pervasive form of ALAN is skyglow, the diffuse brightening of the night sky caused by atmospheric light scattering [3]. The intensity and extent of skyglow are strongly influenced by meteorological conditions, especially cloud cover, which can greatly amplify its reach [8,9,10]. Although dimmer than direct light sources, skyglow often exceeds the luminance of natural celestial features [11,12], extending its influence well beyond urban boundaries and creating broad-scale luminance gradients that span urban to rural areas [9,12].

ALAN is transforming nocturnal environments that many species have adapted to over evolutionary timescales [13,14]. The concept of “ecological light pollution” has been introduced to highlight the growing threats faced by nocturnal wildlife [15], with species- and taxon-specific responses to light varying considerably [16,17,18]. For instance, light pollution is a leading factor in the annual mortality of billions of migratory birds due to building collisions and has been shown to interfere with avian migratory behavior [19,20]. Skyglow may draw birds towards suboptimal stopover habitats during migration, creating ecological traps that increase the mortality of birds [21]. ALAN is also linked to insect declines [22,23], raising concerns for migratory species whose abundance and biomass play critical roles in ecosystems [24].

Many insects make complex flight decisions during long-distance migration [25], relying on cues such as celestial information, wind fields, the Earth’s magnetic field, and topographical features [26,27,28,29]. Among these, celestial cues are one of the most extensively studied orientation mechanisms in migratory insects. Diurnal migrants may use time-compensated solar compasses [30,31], while nocturnal insects navigate using stars, the moon, or large-scale sky luminance patterns [32,33,34]. However, these dim celestial cues at night are easily masked by skyglow, which may interfere with orientation and potentially misguide insect migrants [35,36,37]. Light pollution may act as a powerful attractant, reshaping biomass distributions and contributing to navigational failure [38,39,40]. While the dorsal light response is commonly invoked to explain phototaxis [41], recent radar-based studies reveal that the behavioral effects of ALAN are more nuanced and spatially widespread than previously assumed [42]. To date, most studies have focused on insect attraction to direct light sources. Although celestial luminance gradients have been shown to guide orientation in diurnal nonmigratory insects [43], it remains unclear whether nocturnal migrants can detect and respond to broader-scale luminance gradients such as those created by skyglow. Clarifying this issue is critical for understanding how large-scale light pollution shapes migratory behavior and its underlying mechanisms, which is essential for both pest management and the conservation of migratory insects.

The fall armyworm (FAW), *Spodoptera frugiperda* (J. E. Smith, 1797), a globally distributed nocturnal migratory moth native to tropical and subtropical regions of the Americas [44], exhibits remarkable long-range migratory capability. Since its invasion of West Africa and subsequent spread into China [45,46], it has established a unique north–south migratory pattern with distinct adaptive behaviors [47,48], posing a significant threat to agricultural production [49]. To investigate how spatial luminance gradients influence migratory orientation, we combined controlled indoor simulations with field experiments conducted under natural skyglow conditions. In this study, our primary aim was to experimentally determine whether spatial luminance gradients, particularly those associated with skyglow, can influence the directional orientation of FAW.

We hypothesized that such gradients may be perceived as directional information and could influence the moths’ orientation behavior relative to their expected migratory headings [48]. Clarifying these points will enhance our understanding of nocturnal insect orientation under light-polluted skies and highlight the relevance of spatial luminance structure to ecological models of migration, with potential implications for pest management.

## 2. Materials and Methods

### 2.1. Collection and Rearing of FAW

Late-instar (5th or 6th instar) larvae of the FAW were collected from multiple maize fields in Yuanjiang County, Yunnan Province, China (elevation: 423 m; 23.604° N, 101.977° E). Each larva was reared individually in a transparent plastic tube at the insect-rearing room of the Yuanjiang County Plant Protection Station. Fresh maize leaves were supplied daily until pupation. The rearing room was naturally ventilated, free of active electronic devices, and exposed to ambient daylight, allowing light intensity, photoperiod, and temperature to closely match outdoor conditions. Pupae were transferred to individual transparent plastic cups with a moistened cotton ball to maintain humidity until adult emergence. The day of adult emergence was designated as day 1. Adults were provided with a cotton pad soaked in 10% honey solution and used in orientation experiments at two days of age. Both unmated males and females were used in the orientation experiments. The sex of each individual moth was recorded upon emergence (Tables S1–S3). Because all moths were reared individually and tested two days after emergence, before the typical onset of mating, each individual was considered to be in a pre-reproductive stage at the time of testing. The autumn population collected in September-October was tested under field conditions, as individuals of this cohort are normally expected to orient along a seasonal migratory route [48]. However, these moths did not exhibit the expected migratory heading. This unexpected pattern raised the question of whether skyglow-induced luminance gradients might influence orientation by being interpreted as directional information. To examine this possibility under conditions without seasonal directional bias, we used the non-migratory summer population in controlled indoor experiments. Because this population lacks a consistent seasonal orientation tendency [48], it provided an appropriate context for testing whether luminance gradients alone could act as directional cues. We present the autumn field results first because the phenomenon was initially observed in the spring population, but sky luminance gradients were not recorded during that period. The field experiments were therefore repeated in autumn, when both the migratory population and skyglow conditions could be measured reliably. To maintain a consistent and logical progression, the validated field observations from the autumn generation are shown first, followed by the indoor experiments designed to test the influence of luminance gradients under controlled conditions.

### 2.2. Field Orientation Experiments

Field experiments were conducted to assess the orientation behavior of fall armyworm (FAW) under natural skyglow-induced luminance gradients. The study site was located approximately 1.5 km from Zhega Village and 10.4 km from the urban center of Yuanjiang County, Yunnan Province (23.531° N, 102.077° E; elevation 540 m). Weather conditions were recorded daily. Each evening, the experimental setup was established before sunset to allow moths to acclimate to ambient conditions and experience the gradual onset of nighttime sky luminance. Orientation trials began after complete astronomical nightfall.

Adult moths were tethered at the junction of the thorax and abdomen using a fine copper hook and mounted onto a custom-built flight simulator [48]. Each individual was placed inside a cylindrical arena constructed from homogeneous opaque acrylic (outer diameter: 400 mm; height: 400 mm; wall thickness: 9 mm), which allowed tethered moths to rotate freely and maintain stable flight. The opaque walls of the arena restricted the moth’s visual field exclusively to the zenithal sky, thereby eliminating surrounding landscape features and ground cues as potential orientation references. A transparent acrylic panel (Polymethyl methacrylate; diameter: 500 mm; thickness: 4 mm) was placed above the arena to prevent wind disturbance and stabilize the flight environment, while simultaneously preserving an unobstructed view of the sky for visual orientation. Each moth was allowed to fly freely for 5 min, and its heading was continuously recorded using behavioral tracking software *Flash Flight Simulator Data Acquisition System V1.0* developed by Hui Chen for subsequent analysis. Field experiments were conducted from late September to early November. A total of 25 moths were tested under natural skyglow conditions at new moon.

### 2.3. Laboratory Orientation Experiments

Indoor orientation experiments were conducted in a light-isolated room at the Yuanjiang County Plant Protection Station, free from external illumination and electromagnetic interference. Experiments were initiated approximately 30 min after sunset each day. Prior to testing, each adult moth was allowed to acclimate to the ambient light conditions of the room for 5 min before the start of the experiment.

The experimental apparatus was identical to that used in field trials. Moths were tethered at the junction between the thorax and abdomen to a suspended brass ring and mounted in a flight simulator placed within a cylindrical acrylic flight arena (outer diameter 400 mm, height 400 mm, wall thickness 9 mm). To simulate a luminance gradient, a circular frosted acrylic panel (diameter: 500 mm; thickness: 4 mm) was printed with a graded layer of black paint, producing a continuous decrease in transmittance from left to right above the arena. The direction of the luminance gradient was manipulated by rotating the panel, with the darker end facing either east or west. In control trials, a uniformly frosted milky-white acrylic panel (diameter: 500 mm; thickness: 4 mm) of identical size was used. The center of the overhead panel was aligned with the center of the arena in all trials. The indoor experiment used a full-spectrum light, enclosed in a 90 cm diameter GODOX softbox to ensure even distribution of light within the experimental arena as described in our previous work [27]. Each moth was allowed to fly freely for 5 min while its heading was continuously recorded using the same behavioral tracking software in field orientation experiments for subsequent analysis. To minimize potential carryover effects from gradient exposure, the uniform-luminance control panel was always conducted first. Subsequently, the two gradient panel conditions (darker side facing magnetic east, and darker side facing magnetic west) were presented in randomized order to eliminate order effects. Indoor experiments were conducted in August, with 34 moths tested under each gradient condition and 23 moths under the control condition. The uniform-luminance control group, however, included only 23 moths, as several individuals stopped flying prematurely due to the absence of visual reference cues, resulting in the exclusion of incomplete flight data.

### 2.4. Sky Luminance Gradient Recording

To characterize the spatial luminance structure of the night sky caused by urban skyglow, we recorded panoramic sky images in conjunction with field behavioral trials. A Nikon D610 digital camera (Nikon Corp., Tokyo, Japan) fitted with a Sigma 8 mm F3.5 EX DG fisheye lens (Sigma Corp., Hiroshima, Japan) was used to capture full-sky hemispherical images in RAW format at the experimental site. Exposure bracketing was employed to ensure a high dynamic range, allowing the detection of subtle gradients across a broad spectrum of sky brightness levels. The resulting images were used to generate relative brightness heatmaps for analysis of luminance distribution and correlation with insect orientation.

### 2.5. Sky Measurement and Visualization of Light–Dark Contrast

On each experimental night, three sets of exposure-bracketed images were captured at the same time using a digital camera fitted with a fisheye lens (ISO speed 400, f/3.5, exposure times: 10 s, 30 s, and 2 min) [6]. Images were taken with the camera oriented vertically toward the zenith, positioned at the northern edge of the arena at a height of 1.6 m above ground level. Care was taken to exclude local vegetation and experimenters from the field of view. At rural sites where space allowed, the camera and tripod were relocated 4 m from the arena during trials to avoid obstructing the moth’ view of the sky.

To visualize the light–dark contrast in the panoramic sky, exposure-bracketed images were merged into high dynamic range (HDR) images using the “Merge to HDR Pro” function in Adobe Photoshop (version 24.0.0). The resulting HDR images were exported as TIFF files and processed with a custom R script. RGB values were extracted pixel by pixel, and grayscale luminance values were computed for each pixel using a moth-weighted channel ratio (R:G:B = 0:3.33:6.67), based on known armyworm moth photoreceptor sensitivities [50]. These values were then used to generate heatmaps, providing a spatial visualization of luminance gradients across the night sky.

### 2.6. Experimental Lighting and Luminance Quantification

**Figure A1:**
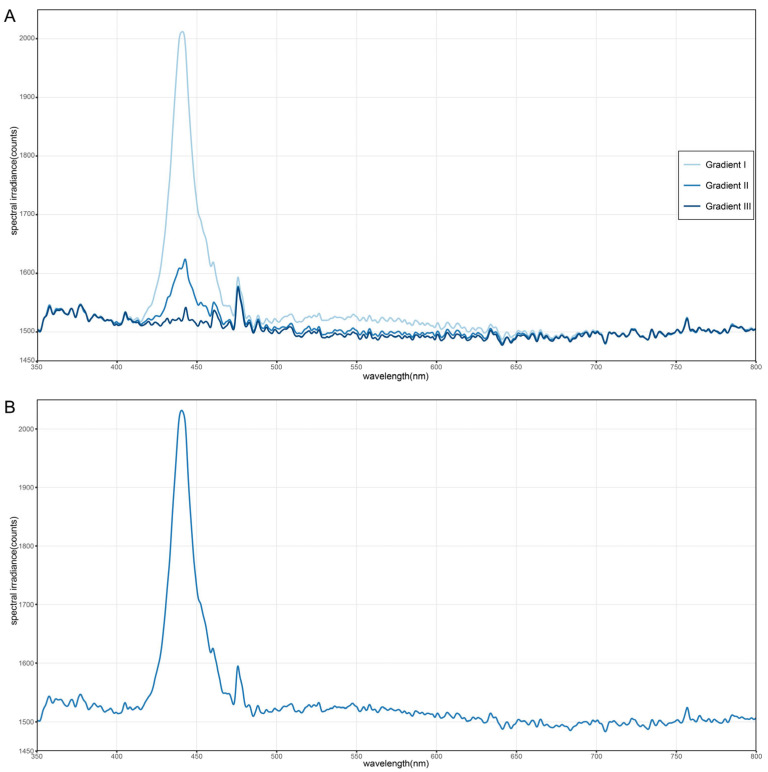
Quantification of the relative luminance gradient in laboratory experiments. (**A**). Spectral irradiance of the gradient panel. Relative spectral irradiance measurements (in counts) were taken at three positions on the custom-printed acrylic gradient panel under the full-spectrum laboratory light source: Gradient I (the brightest end), Gradient II (the midpoint), and Gradient III (the darkest end). The data demonstrate that the acrylic panel generated a continuous luminance gradient, characterized by three distinct intensity levels at 440 nm, while simultaneously maintaining a constant spectral composition across the panel (identical peak wavelength and spectral shape). (**B**). Spectral irradiance of the uniformly frosted milky-white acrylic panel. The blue line represents the relative spectral irradiance (counts) measured at the center of the uniformly frosted milky-white acrylic panel, illustrating the spectral composition of the illumination.

To verify and quantify the luminance gradient produced by the custom-printed acrylic panel (described in Section 2.3), the light intensity was measured under experimental lighting conditions. We utilized a ATP5020R spectrometer (Optosky (Xiamen) Photonics Inc., Xiamen, China) to record the relative light output directly below the overhead panel at three critical positions: the brightest end, the midpoint, and the darkest end of the gradient. To achieve a high signal-to-noise ratio, the data acquisition parameters were set as follows: the integration time was 2500 ms, and 3 scans were averaged for each measurement. Furthermore, 20 cycles of smoothing were applied during post-processing to minimize random noise. The fiber optic probe of the instrument was placed at the height of the tethered moth and oriented vertically upward toward the panel to accurately simulate the visual input perceived by the insect. Simultaneously, measurements were also taken under the uniform-luminance control panel to establish the baseline light intensity. The resulting quantitative data (measured in counts, representing relative light intensity) were used to confirm the existence and magnitude of the continuous luminance gradient that the moths were exposed to, thereby validating the experimental manipulation. Luminance measurement results are detailed in Figure A1.

### 2.7. Quantification and Statistical Analysis

All statistical analyses and data visualizations were conducted in R (version 4.3.2; https://www.r-project.org/, accessed on 31 October 2023).

#### 2.7.1. Analysis of Orientation Behavior

Moth orientation behavior was analyzed using a custom R script implementing the Moore Modified Rayleigh Test (MMRT), combined with bootstrap-based confidence interval estimation. While the standard Rayleigh test assesses whether directional data deviate from a uniform distribution, it assumes equal orientation strength across individuals and does not account for differences in directional certainty. In contrast, MMRT incorporates inter-individual variation by applying a rank-weighted, nonparametric approach that improves sensitivity in behavioral studies with variable orientation strengths. Each individual’s orientation was represented as a vector on a polar plot, where the angle corresponds to the mean flight direction and the length indicates orientation strength (*r* value, Tables S1–S3), calculated using the circular package in R. The *r* value ranges from 0 (random or highly variable heading) to 1 (perfect directional consistency). These *r* values were then used to compute a rank-weighted mean vector (MV) and the MMRT test statistic *R*, which serves as a population-level measure of orientation strength. Because *R* is sensitive to sample size, bootstrap resampling was applied to generate robust confidence intervals for statistical inference. Detailed statistical results and statistical results for MMRT data are presented in Table A1.

#### 2.7.2. Analysis of Flight Stability

To evaluate both directional tendency and flight stability of individual moths under different treatments, we used two primary metrics: the Rayleigh test *r* value and the mean angular change per second. Directional orientation was assessed based on the Rayleigh test applied to each individual’s flight direction. The *r* value, which ranges from 0 to 1, indicates the strength of orientation, with higher values indicating stronger directional consistency. To quantify flight stability, we calculated the mean circular change per second by measuring the absolute value of the circular difference in flight angle between consecutive one-second intervals. This metric served as an indicator of the consistency and stability of flight orientation.

**Figure A2:**
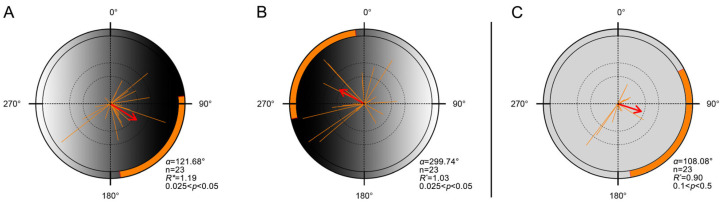
Laboratory orientation of FAW moths in response to simulated luminance gradients. (**A**,**B**). Orientation distributions of moths under a luminance gradient created using a frosted gradient acrylic panel with a black-to-white transition. (**A**). The darker end of the panel faced magnetic east (n = 23, α = 121.68°, 95% CI = 83.99–172.25°, R* = 1.19, *p* < 0.05). (**B**). The panel was rotated 180°, placing the darker end to the magnetic west (n = 23; α = 299.74°; 95% CI = 258.16–353.15°; R* = 1.03; *p* < 0.05). (**C**). Orientation directions of moths under control conditions using a uniformly frosted (non-gradient) acrylic panel (n = 23, α = 108.08°, 95% CI = 61.58–170.22°, R* = 0.90, *p* > 0.1). Each orange line represents the individual mean vector (*r*). Each red arrow represents the population mean vector (MV), and orange shaded arcs denote its 95% CI. All other definitions as in Figure 1. Detailed statistical results are provided in Table A1.

The same cohort of 23 individuals participated in all indoor treatment phases (uniform control and the two gradient orientations). Specific orientation results are presented in Figure A2. We therefore employed repeated-measures non-parametric tests to analyze differences in both the ***r*** value and the mean angular change among the treatment groups. We first used the Friedman test to determine whether overall differences existed among treatment conditions for each metric. When significant effects were detected (*p* < 0.05), pairwise comparisons were subsequently performed using the Wilcoxon signed-rank test (paired). The resulting *p*-values were corrected using the Benjamini–Hochberg (BH) procedure to control for the false discovery rate. Groups labeled with different letters were considered significantly different (*p* < 0.05). Detailed statistical results for directional strength and flight stability are presented in Table A2 and Table A3, respectively.

## 3. Results

### 3.1. Skyglow-Induced Luminance Gradients Affect Orientation in the Field

**Figure 1:**
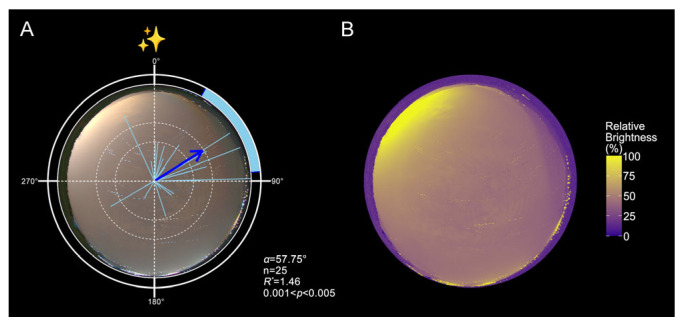
Field orientation of FAW in response to urban skyglow-induced luminance gradients. (**A**). Orientation distributions of moths under moonless nights (n = 25, *α* = 57.75°, 95% CI = 28.59–84.45°, R* = 1.46, *p* < 0.05). (**B**). Stacked relative luminance heatmaps of the moonless night skies. In panel A, each light-blue line represents the individual mean vector (*r*), ranging from 0 to 1, with the outer edge of the plot corresponding to *r* = 1. Each blue arrow represents the population mean vector (MV, *α*), calculated using Moore’s Modified Rayleigh Test (MMRT), which accounts for both the direction and strength of individual orientation responses. Arrow length reflects the R* value, indicating the concentration of individual headings around the mean direction. Dashed circles denote *R* thresholds corresponding to *p* < 0.05 and *p* < 0.01. The blue shaded arc indicates the 95% CI for the mean vector. The circular plot labels 0°, 90°, 180°, and 270° correspond to magnetic north, east, south, and west, respectively. Purple regions indicate areas of lowest relative luminance, while yellow regions denote the brightest portions of the sky. Detailed statistical results are provided in Table A1.

Under natural sky luminance gradients, moths exhibited a consistent northeastward orientation on moonless nights (Figure 1A, Table A1; n = 25; *α* = 57.75°). This directional preference corresponded with the darker portion of the sky, aligned with the direction of the Yunnan-Guizhou Plateau and opposite major sources of light pollution. Composite heatmaps generated from stacked panoramic images confirmed the presence of a consistent sky luminance gradient across conditions (Figure 1B). Notably, the observed orientation toward the darkest sky region contrasted with the expected southward seasonal migratory direction during autumn [48].

### 3.2. Indoor Luminance Gradient Experiments Replicate Field-Based Orientation Behavior

To isolate the mechanism and confirm the use of luminance gradients as directional cues, we tested non-migratory summer-generation adults in a controlled indoor flight simulator.

**Figure 2:**
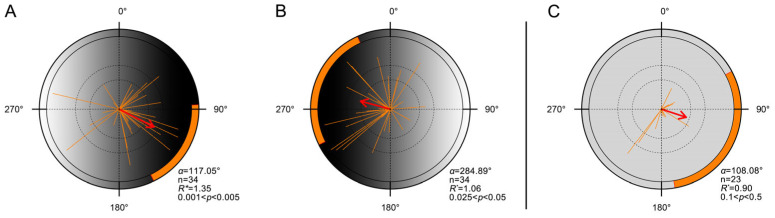
Laboratory orientation of FAW moths in response to simulated luminance gradients. (**A**,**B**). Orientation distributions of moths under a luminance gradient created using a frosted gradient acrylic panel with a black-to-white transition. (**A**). The darker end of the panel faced magnetic east (n = 34, *α* = 117.05°, 95% CI = 86.69–154.19°, R* = 1.35, *p* < 0.05). (**B**). The panel was rotated 180°, placing the darker end to the magnetic west (n = 34; *α* = 284.89°; 95% CI = 243.79–335.72°; R* = 1.06; *p* < 0.05). (**C**). Orientation directions of moths under control conditions using a uniformly frosted (non-gradient) acrylic panel (n = 23, *α* = 108.08°, 95% CI = 61.58–170.22°, R* = 0.90, *p* > 0.1). Each orange line represents the individual mean vector (*r*). Each red arrow represents the population mean vector (MV), and orange shaded arcs denote its 95% CI. All other definitions as in Figure 1. Detailed statistical results are provided in Table A1.

Moths exhibited a clear directional preference toward the darker region of the gradient. When the dark area was placed in the magnetic east, individuals oriented eastward (Figure 2A, Table A1; n = 34; *α* = 117.05°); when reversed, they oriented westward (Figure 2B, Table A1; n = 34; *α* = 284.89°). In contrast, under the uniform control panel, flight was generally unstable and no significant group orientation was observed (Figure 2C, Table A1; n = 23; *α* = 108.08°), consistent with the absence of collective orientation previously reported under summer-like photoperiods [48].

**Figure 3:**
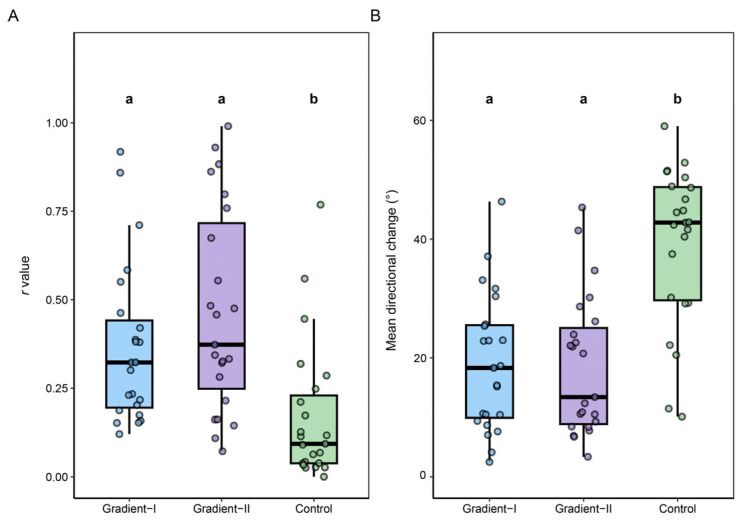
Flight stability of FAW under different luminance gradient treatments. (**A**). Distribution of Rayleigh *r* values for individual moth orientation across the three treatment groups. (**B**). Distribution of the mean directional (circular) change per second for the same individuals, reflecting flight stability. The treatment order (Gradient-I, Gradient-II, Control) matches the ‘A’, ‘B’, and ‘C’ conditions in Figure 2, respectively. Because all 23 individuals participated in every treatment, differences among groups were evaluated using repeated-measures non-parametric tests. Overall effects were first assessed with the Friedman test. When significant differences were detected (*p* < 0.05), pairwise comparisons were performed using the Wilcoxon signed-rank test, with *p*-values adjusted using the Benjamini–Hochberg (BH) procedure to control the false discovery rate. Groups sharing the same letter do not differ significantly; groups with different letters are significantly different (*p* < 0.05). Box plots show the interquartile range (IQR), the median (horizontal line), and whiskers extending to 1.5× IQR. Detailed statistics for *r* values and directional stability are provided in Table A2 and Table A3.

The individual directional strength, quantified by the Rayleigh *r* value, varied significantly across the three treatment conditions in the 23 moths tested across all phases (Friedman test, *p* < 0.01). Individuals showed a significantly higher *r* value under both gradient panel conditions (Gradient-I and Gradient-II) compared to the uniform control panel, with no significant difference found between the two gradient orientations (Figure 3A, Table A2; Paired Wilcoxon signed-rank tests).

To further assess flight consistency, we quantified the mean angular change per second. This metric also varied significantly across treatments (Friedman test, *p* < 0.001). Moths exhibited significantly lower angular change under both gradient panel conditions compared to the uniform control, indicating more stable flight. There was no significant difference in angular change between the two gradient orientations (Figure 3B, Table A3; Paired Wilcoxon signed-rank tests).

These results suggest that the illuminance gradient promoted more stable flight and supported strong, consistent directional responses in individual moths, confirming that luminance gradients can function as a potent directional cue, even in a population that typically lacks group-level seasonal orientation.

## 4. Discussion

Among the most pervasive and insidious forms of ALAN is skyglow—the diffuse brightening of the night sky—casting an invisible yet far-reaching ecological net over natural landscapes [3,9,12,51]. Our findings demonstrate that skyglow-induced luminance 51 gradients can significantly influence the orientation behavior of a nocturnally migrating moth. Across both indoor simulations and natural skyglow conditions, FAW individuals consistently deviated from their expected seasonal heading patterns and instead oriented toward darker regions of their visual field. This pattern was not random but highly stable, suggesting that moths can detect and respond to spatial asymmetries in luminance at broader spatial scales.

Our results demonstrate that luminance gradients, such as those formed by skyglow, may serve as directional cues that interfere with the innate orientation behavior of nocturnally migrating moths. Unlike attraction to bright points of light, this response appears to involve a repulsion from brighter regions, reminiscent of the celestial navigation strategies of other species but operating in the opposite direction: while dung beetles use skylight intensity gradients to orient toward brighter sectors [43,52,53], FAWs orient consistently away from them. This may reflect an evolved strategy to favor darker sky sectors under natural celestial conditions, where clearer views of the night sky typically correspond to migratory-relevant cues [35]. Furthermore, this bias toward darker sky sectors might offer an immediate survival benefit by mitigating predation risk from visually dependent insectivores. Studies confirm that skyglow effectively relieves crepuscular birds from the natural visual constraints of dim light, facilitating prey detection and consequently extending their foraging activity later into the night [54,55]. Such a tendency could also align with recent evidence suggesting that light attraction in moths is declining as a consequence of its detrimental effects on survival [56]. Maintaining a dark-adapted state may be functionally advantageous for celestial orientation, as it enhances light sensitivity and improves contrast detection under dim conditions [35,57,58]. Notably, dung beetles rolling toward a light source do so backwards, positioning their body and dung ball between their eyes and the light, which may serve a similar purpose. Likewise, a moth flying away from a light source may effectively shade its eyes with its thorax, preserving the visual sensitivity needed for stable orientation. In light-polluted environments, however, this behavioral tendency may become counterproductive, leading to orientation away from optimal routes and potentially transforming skyglow may function as a form of ecological trap [6,21,39]. Although our ground-based findings focus on initial orientation, misalignment caused by luminance gradients at takeoff could translate into suboptimal large-scale windborne trajectories and ultimately contribute to migratory failure at high altitudes [28,29].

Our findings thus expand the framework of ALAN impacts by identifying spatial luminance gradients as an additional directional cue influencing nocturnal migration. While much of the current focus has centered on temporal disruptions (e.g., circadian desynchrony or phototaxis toward point sources) [22,59], our results indicate that spatial luminance structure can also affect orientation behavior. These gradients should be incorporated into mechanistic models of nocturnal animal navigation and environmental risk.

While our study provides clear evidence that skyglow-induced luminance gradients influence orientation behavior in migratory moths, several questions remain open. First, the absolute luminance and spectral composition of the natural sky were not quantified in field trials [60,61], limiting the ability to generalize luminance thresholds across environments. Second, our findings are based on classical flight simulator assays and therefore do not represent long-range orientation in free-flying individuals [62], nor do they reveal how luminance-gradient cues interact with other navigational systems across insect taxa [30,32,33,34,62]. Third, since sex- and age-related variation was not assessed here, potential sex- or age-specific differences in luminance-gradient responses remain unknown. Additionally, the field component of our study was limited by a relatively small sample size, as trials could only be conducted under natural skyglow during the new-moon period. As a result, the field data primarily describe the behavior of one population under a specific seasonal and skyglow context. Further work incorporating larger sample sizes, multiple populations across different migratory seasons, direct measurements of sky luminance, and tracking of free-flying individuals will be essential for assessing the generality, ecological relevance, and underlying mechanisms of luminance-gradient-guided orientation.

## 5. Conclusions

Our study reveals that skyglow, a pervasive form of ALAN, can significantly bias the instantaneous orientation of nocturnal FAWs. Individuals consistently oriented toward the darker region of luminance gradients under both indoor and field conditions, indicating that such gradients can serve as powerful visual references for short-term orientation. As natural celestial cues become increasingly obscured by diffuse urban light, these luminance gradients may function as alternative visual orientation cues, potentially misleading nocturnal insects. Although our experiments do not assess migratory trajectories or changes in the routes taken by free-flying moths, the observed orientation bias indicates that skyglow, independent of discrete light sources, can influence short-term visual orientation. Together, these findings provide behavioral evidence that luminance gradients represent an underrecognized component of light pollution, with important implications for nocturnal insect navigation and movement ecology.

## Data Availability

The original contributions presented in this study are included in the article/Supplementary Material. Further inquiries can be directed to the corresponding author.

## Supplementary Materials

The following supporting information can be downloaded at: https://www.mdpi.com/article/10.3390/insects16121252/s1. Table S1. Individual Rayleigh test data for the field experiments. Table S2. Individual Rayleigh test data for the indoor experiments. Table S3. Individual Rayleigh test data for the indoor experiments completing all 3 treatments.

## Abbreviations

The following abbreviations are used in this manuscript:

ALAN	Artificial Light at Night
FAW	Fall Armyworm
MV	Mean Vector
MMRT	Moore’s Modified Rayleigh Test
IQR	Interquartile Range
BH	Benjamini–Hochberg

## Appendix A

**Table A1:** Detailed data of flight orientation behavior assay.

Group	Treatment	MV	*R**	n	*p* Value
Field Experiments	Starry nights	57.75	1.46	25	0.001 < *p* < 0.005
Indoor Experiments	Gradient-I	117.05	1.35	34	0.001 < *p* < 0.005
		121.68	1.19	23	0.025 < *p* < 0.05
	Gradient-II	284.89	1.06	34	0.025 < *p* < 0.05
		299.74	1.03	23	0.025 < *p* < 0.05
	Control	108.08	0.90	23	0.1 < *p* < 0.5

Note: In the indoor experiments, the treatment order (Gradient-I, Gradient-II, Control) matches the ‘A’, ‘B’, and ‘C’ conditions in Figure 2.

**Table A2:** Multiple comparison results of the mean Rayleigh’s *r* values of indoor experiments across different treatment conditions, derived from the Friedman test followed by paired Wilcoxon signed-rank tests. *p*-values were adjusted using the Benjamini–Hochberg (BH) procedure to control for the false discovery rate.

Comparison Group	*W* Value	BH-Corrected *p* Value	Median Difference
A−B	103	0.30	−0.05
A−C	234	0.0036	0.23
B−C	239	0.0036	0.28

Note: Comparison groups (A, B, C) are defined as follows, consistent with the treatments described in Table A1: A: Gradient-I; B: Gradient-II; C: Control.

**Table A3:** Multiple comparison results for the mean angular change per second (flight stability) of indoor experiments across different treatment conditions, derived from the Friedman test followed by paired Wilcoxon signed-rank tests. *p*-values were adjusted using the Benjamini–Hochberg (BH) procedure to control for the false discovery rate.

Comparison Group	*W* Value	BH-Corrected *p* Value	Median Difference
A−B	161	0.50	4.92
A−C	17	0.00014	−24.47
B−C	22	0.00019	−29.39

Note: Comparison groups (A, B, C) are defined as follows, consistent with the treatments described in Table A1: A: Gradient-I; B: Gradient-II; C: Control.
