# Supplementary material for: Skyglow-Induced Luminance Gradients Influence Orientation in a Migratory Moth
Source: Insects. 2025 Dec 10;16(12):1252. doi: 10.3390/insects16121252 (PMC12734188; doi:10.3390/insects16121252)
Supplement: Supplementary file 1 [file insects-16-01252-s001.zip › insects-3998576-supplementary.pdf]

Table S1. Individual Rayleigh test data for the field experiments.

Mean direction (°)	*r*	Sex	Treatment
1.448090032	0.3869892	male	Starry nights
74.86409421	0.3532289	male	Starry nights
123.1748139	0.2429158	male	Starry nights
15.71603778	0.3781391	male	Starry nights
123.1573726	0.0722307	female	Starry nights
241.6509763	0.1117307	male	Starry nights
141.6135056	0.1072109	male	Starry nights
74.51752689	0.5590194	female	Starry nights
68.68534135	0.951684	female	Starry nights
34.37834852	0.4520906	female	Starry nights
130.0601579	0.2281483	female	Starry nights
60.33185474	0.6222142	female	Starry nights
210.5889558	0.2039649	female	Starry nights
88.5449132	0.9954948	female	Starry nights
55.05681538	0.1390681	female	Starry nights
89.28972038	0.4852583	female	Starry nights
57.5568333	0.9231163	female	Starry nights
335.1776364	0.7462932	female	Starry nights
239.7515745	0.5234358	female	Starry nights
350.3543881	0.2074732	female	Starry nights
5.149951551	0.3984643	female	Starry nights
89.03147162	0.17774	female	Starry nights
37.24043546	0.1773128	female	Starry nights
162.1403887	0.3886021	male	Starry nights
21.3804086	0.2469774	male	Starry nights

Table S2. Individual Rayleigh test data for the indoor experiments.

Mean direction (°)	*r*	Sex	Treatment
131.5762988	0.3799266	female	Gradient-I
144.784812	0.2172228	male	Gradient-I

152.010181	0.3872883	male	Gradient-I
108.7928127	0.8589352	female	Gradient-I
199.1094342	0.2336124	female	Gradient-I
50.62076143	0.7108948	female	Gradient-I
131.1003259	0.1521881	female	Gradient-I
62.99046303	0.4204941	female	Gradient-I
128.532926	0.1580239	male	Gradient-I
54.3793547	0.3225993	male	Gradient-I
140.3031146	0.2023273	female	Gradient-I
106.9148214	0.3011548	female	Gradient-I
97.97855094	0.1741755	female	Gradient-I
26.82358209	0.3812013	female	Gradient-I
28.16288045	0.1874992	female	Gradient-I
167.9467728	0.5507516	female	Gradient-I
318.5987984	0.1204557	female	Gradient-I
231.9624699	0.9178685	female	Gradient-I
81.06317458	0.5840029	female	Gradient-I
149.345891	0.3229097	male	Gradient-I
258.5728422	0.2306226	male	Gradient-I
118.5863879	0.1526884	female	Gradient-I
241.1117899	0.4628331	female	Gradient-I
168.763634	0.7864982	female	Gradient-I
35.80157091	0.3324386	female	Gradient-I
116.7413674	0.5255628	female	Gradient-I
121.5243117	0.6724368	female	Gradient-I
102.3839667	0.5140498	female	Gradient-I
115.4592984	0.8989214	female	Gradient-I
327.3512269	0.3990327	female	Gradient-I
283.2821971	0.9414519	female	Gradient-I
128.6811624	0.3093372	female	Gradient-I
46.71801235	0.5273327	male	Gradient-I
128.6705535	0.8980185	male	Gradient-I

317.4883024	0.8828543	female	Gradient-II
256.5039623	0.9299734	male	Gradient-II
229.5667255	0.8619649	male	Gradient-II
272.9047658	0.1615084	female	Gradient-II
86.74234832	0.0717399	female	Gradient-II
356.1957602	0.4577533	female	Gradient-II
119.1483305	0.3215215	female	Gradient-II
148.5278358	0.332678	female	Gradient-II
32.85964612	0.7587163	male	Gradient-II
305.4461515	0.3730195	male	Gradient-II
296.770747	0.6746779	female	Gradient-II
355.3927219	0.3436414	female	Gradient-II
66.93201149	0.1087607	female	Gradient-II
317.9747583	0.7978989	female	Gradient-II
265.3600854	0.3259206	female	Gradient-II
226.2136424	0.1617731	female	Gradient-II
235.6657587	0.9905178	female	Gradient-II
263.5090586	0.4753682	female	Gradient-II
60.11261854	0.2040896	female	Gradient-II
15.22609815	0.5541749	male	Gradient-II
238.4374243	0.1445568	male	Gradient-II
86.03761759	0.4829096	female	Gradient-II
301.465484	0.2149161	female	Gradient-II
118.7583802	0.2819273	female	Gradient-II
244.4054353	0.0719336	female	Gradient-II
195.7162829	0.3154762	female	Gradient-II
13.13335798	0.4471982	female	Gradient-II
183.0052078	0.3206501	female	Gradient-II
168.5210115	0.0237279	female	Gradient-II
346.0737563	0.7393239	female	Gradient-II
202.4104696	0.6500165	female	Gradient-II
357.1588616	0.1816466	female	Gradient-II

232.9641681	0.9346989	male	Gradient-II
248.4238776	0.6582473	male	Gradient-II
84.31496035	0.0380575	female	Control
67.63638242	0.0679006	male	Control
66.43322727	0.1730071	male	Control
72.66938962	0.026334	female	Control
62.95312614	0.2111301	female	Control
12.30481818	0.0004043	female	Control
15.11012637	0.0419008	female	Control
144.4701695	0.0269305	female	Control
122.1870158	0.4460856	male	Control
190.469783	0.0253919	male	Control
87.96971346	0.090183	female	Control
25.83063331	0.2481548	female	Control
26.06509608	0.3189152	female	Control
255.0510399	0.0374208	female	Control
65.75629703	0.0630751	female	Control
171.8244181	0.0928839	female	Control
355.7143525	0.0329123	female	Control
158.8761275	0.1138458	female	Control
197.1515642	0.2858748	female	Control
150.5615826	0.126702	male	Control
214.3647849	0.5597162	male	Control
58.14710352	0.1171533	female	Control
217.3984956	0.7684012	female	Control

Table S3. Individual Rayleigh test data for the indoor experiments completing all 3 treatments.

**Mean direction (°)**	***r***	**Sex**	**Treatment**
131.5762988	0.3799266	female	Gradient-I
144.784812	0.2172228	male	Gradient-I
152.010181	0.3872883	male	Gradient-I
108.7928127	0.8589352	female	Gradient-I

199.1094342	0.2336124	female	Gradient-I
50.62076143	0.7108948	female	Gradient-I
131.1003259	0.1521881	female	Gradient-I
62.99046303	0.4204941	female	Gradient-I
128.532926	0.1580239	male	Gradient-I
54.3793547	0.3225993	male	Gradient-I
140.3031146	0.2023273	female	Gradient-I
106.9148214	0.3011548	female	Gradient-I
97.97855094	0.1741755	female	Gradient-I
26.82358209	0.3812013	female	Gradient-I
28.16288045	0.1874992	female	Gradient-I
167.9467728	0.5507516	female	Gradient-I
318.5987984	0.1204557	female	Gradient-I
231.9624699	0.9178685	female	Gradient-I
81.06317458	0.5840029	female	Gradient-I
149.345891	0.3229097	male	Gradient-I
258.5728422	0.2306226	male	Gradient-I
118.5863879	0.1526884	female	Gradient-I
241.1117899	0.4628331	female	Gradient-I
317.4883024	0.8828543	female	Gradient-II
256.5039623	0.9299734	male	Gradient-II
229.5667255	0.8619649	male	Gradient-II
272.9047658	0.1615084	female	Gradient-II
86.74234832	0.0717399	female	Gradient-II
356.1957602	0.4577533	female	Gradient-II
119.1483305	0.3215215	female	Gradient-II
148.5278358	0.332678	female	Gradient-II
32.85964612	0.7587163	male	Gradient-II
305.4461515	0.3730195	male	Gradient-II
296.770747	0.6746779	female	Gradient-II
355.3927219	0.3436414	female	Gradient-II
66.93201149	0.1087607	female	Gradient-II

317.9747583	0.7978989	female	Gradient-II
265.3600854	0.3259206	female	Gradient-II
226.2136424	0.1617731	female	Gradient-II
235.6657587	0.9905178	female	Gradient-II
263.5090586	0.4753682	female	Gradient-II
60.11261854	0.2040896	female	Gradient-II
15.22609815	0.5541749	male	Gradient-II
238.4374243	0.1445568	male	Gradient-II
86.03761759	0.4829096	female	Gradient-II
301.465484	0.2149161	female	Gradient-II
84.31496035	0.0380575	female	Control
67.63638242	0.0679006	male	Control
66.43322727	0.1730071	male	Control
72.66938962	0.026334	female	Control
62.95312614	0.2111301	female	Control
12.30481818	0.0004043	female	Control
15.11012637	0.0419008	female	Control
144.4701695	0.0269305	female	Control
122.1870158	0.4460856	male	Control
190.469783	0.0253919	male	Control
87.96971346	0.090183	female	Control
25.83063331	0.2481548	female	Control
26.06509608	0.3189152	female	Control
255.0510399	0.0374208	female	Control
65.75629703	0.0630751	female	Control
171.8244181	0.0928839	female	Control
355.7143525	0.0329123	female	Control
158.8761275	0.1138458	female	Control
197.1515642	0.2858748	female	Control
150.5615826	0.126702	male	Control
214.3647849	0.5597162	male	Control
58.14710352	0.1171533	female	Control

217.3984956	0.7684012	female	Control

Note: Moths completing all 3 treatments, n=23.
